# The Uniaxial Stress–Strain Relationship of Hyperelastic Material Models of Rubber Cracks in the Platens of Papermaking Machines Based on Nonlinear Strain and Stress Measurements with the Finite Element Method

**DOI:** 10.3390/ma14247534

**Published:** 2021-12-08

**Authors:** Huu-Dien Nguyen, Shyh-Chour Huang

**Affiliations:** 1 Department of Mechanical Engineering, National Kaohsiung University of Science and Technology, No.415, Jiangong Rd., Sanmin Dist., Kaohsiung City 807618, Taiwan; nh.dien@hutech.edu.vn; 2 Institute of Engineering, Ho Chi Minh City University of Technology—HUTECH, No.475A, Dien Bien Phu Rd., Binh Thanh Dist., Ho Chi Minh City 700000, Vietnam

**Keywords:** finite element method, hyperelastic material, rubber material, interface crack

## Abstract

Finite element analysis is extensively used in the design of rubber products. Rubber products can suffer from large amounts of distortion under working conditions as they are nonlinearly elastic, isotropic, and incompressible materials. Working conditions can vary over a large distortion range, and relate directly to different distortion modes. Hyperelastic material models can describe the observed material behaviour. The goal of this investigation was to understand the stress and relegation fields around the tips of cracks in nearly incompressible, isotropic, hyperelastic accouterments, to directly reveal the uniaxial stress–strain relationship of hyperelastic soft accouterments. Numerical and factual trials showed that measurements of the stress–strain relationship could duly estimate values of nonlinear strain and stress for the neo-Hookean, Yeoh, and Arruda–Boyce hyperelastic material models. Numerical models were constructed using the finite element method. It was found that results concerning strains of 0–20% yielded curvatures that were nearly identical for both the neo-Hookean, and Arruda–Boyce models. We could also see that from the beginning of the test (0–5% strain), the curves produced from our experimental results, alongside those of the neo-Hookean and Arruda–Boyce models were identical. However, the experiment’s curves, alongside those of the Yeoh model, converged at a certain point (30% strain for Pieces No. 1 and 2, and 32% for Piece No. 3). The results showed that these finite element simulations were qualitatively in agreement with the actual experiments. We could also see that the Yeoh models performed better than the neo-Hookean model, and that the neo-Hookean model performed better than the Arruda–Boyce model.

## 1. Introduction

The main goal of this study was the determination of material models, and constants, for the finite element analysis of rubber jounces, which serve as platens in papermaking machines. Such rubber products must fulfill predetermined special requirements. Without considering the effect of time, such rubber-like hyperelastic materials are generally considered to be isotropic and incompressible. Operational circumstances often beget large distortions, and under these conditions, rubber can demonstrate largely nonlinear material properties [1,2,3,4,5].

In service, elastomeric corridors are subjected to severe mechanical loadings that can lead to their rupture. Some forces such as compression and pressure cause wear and tear on rubber cracks. This rupture is associated with the creation and extension of cracks. Indeed, several length scales are involved in the fracture process. For example, the rigidity of similar accoutrements influences their energy release rate, leading to the reduction in crack and cavitation formation in front of the crack tips. Thus, a process-zone bounded by a circle (of radius *r*_c_) should be generated around the crack tip [6,7,8,9].

Developing a new polymer with distinct properties is necessary to overcome these issues. However, the quality of similar polymeric compounds is dependent on the precision with which they can be manufactured, and the processing techniques that are used [10]. The buttressing of polymers with nanoparticles has become an attractive field of nanotechnology, and one that is reserved for only the most advanced polymer manufacturing companies [11]. According to the authors of [12], the buttressing of polymers with carbon nanotubes has become popular, and has been applied in numerous fields such as the engineering of automobiles, wind turbines, aeronautical vehicles, and packaging, so probing their mechanical properties, e.g., their elasticity, has attracted the attention of numerous researchers [13,14].

A literature overview of linearly elastic materials provided several techniques for the evaluation and prediction of elastostatic fields, such as eigenfunction expansion systems, complex implicit systems, and integral transform systems. Among the techniques used to dissect the problems associated with cracks, the distributed disturbance approach is an effective means of treatment. Lately, the flash dynamic–stress intensity factors (DIFs) for multiple cracks have been investigated for the fabrics of functionally graded materials [15], e.g., interface cracks in double-clicking facets, and piezoelectric–piezomagnetic mixtures. In these papers, integral transformations are used alongside the distributed disturbance approach, and the numerical inversion system, to gain Cauchy singular integral equations that are answered numerically, thereby allowing for the determination of DIFs [16,17].

In the present work, a new approach was proposed to determine the asymptotic stress and relegation fields at the crack tip in misshapen configurations of nearly incompressible rubber accoutrements. We used a native equation for rubber that was grounded on the compressible Ogden model [18]. To the extent of our knowledge, the use of this approach has not previously been reported in the literature for similar accoutrements [19,20].

Our novel approach considers that boundary conditions will be reformulated in misshapen appendages, in discrepancy with their natural state (see Refs. [21,22,23]). The obtained results could be useful for checking the validity of complex numerical schemes, and it was also clear that stress and strain could be used to estimate the value of nonlinear strain in hyperelastic models. In addition, one of the main advantages of the present approach is that it only requires the knowledge of Young’s modulus and Poisson’s ratio to decipher the stresses and relegation fields near the tip of a crack. Accordingly, the results arising from this approach help in the development of accurate failure criteria, and in rapidly assessing the validity of complex finite element computations [24].

## 2. The Crack Problem

Linear elastic fracture mechanics are grounded on a clear description of the near-crack-tip stress field developed by Irwin. Consider a crack in a horizonless plate, with crack length 2a, and an ever-applied tensile stress acting vertically to the crack plane (mode I). Irwin expressed the near-crack-tip stress field as a series result [19]:

(1) $$\sigma_{ij}\left(r,\theta \right)=\frac{K_{I}}{\sqrt{2\pi r}}f_{ij}\left(\theta \right)+O\left(r^{1/2}\right)+\ldots$$

where $\sigma_{ij}$ is the near-crack-tip stress, $K_{I}$ is the mode *I* stress intensity factor, $K_{I}=\sigma^{\infty }\sqrt{\pi a}$, and (*r*, *θ*) are the spherical polar equivalents of a point with respect to the crack tip. In the near-crack-tip region (*r*→0), and the first term of the result series serves as an acceptable approximation of the elastic stress field for most operations:

(2) $$\sigma_{ij}\left(r,\theta \right)=\frac{K_{I}}{\sqrt{2\pi r}}f_{ij}\left(\theta \right)=\frac{\sigma^{\infty }\sqrt{\pi a}}{\sqrt{2\pi r}}f_{ij}\left(\theta \right)$$

where $\sigma_{ij}$ is the near-crack-tip stress, $K_{I}$ is the mode I stress intensity factor which serves as a scalar multiplier of the crack tip stress field, and $\;\sigma^{\infty }$ is the remotely applied stress.

The variation in the stress *σ* $\sigma_{y}$ as a function of *r* at *θ* = 0. For large values of *r* in Equation (2), $\;\sigma_{y}$ approaches zero, whereas in reality it can only ever fall to the level of applied stress (*σ*∞). As r approaches zero at the crack tip, $\;\sigma_{y}$ approaches infinity. Hence, it is clear that Equation (2) is valid only for a limited region around the crack tip, and is more accurate for low values of $\sigma^{\infty }$.

The direct elastic crack results for stress near the crack tip are [19]:

(3) $$\begin{array}{l} \sigma_{r}=\frac{K_{I}}{\sqrt{2\pi r}}\left(\frac{5}{4}cos\frac{\theta }{2}-\frac{1}{4}cos\frac{3\theta }{2}\right);\\ \sigma_{\theta }=\frac{K_{I}}{\sqrt{2\pi r}}\left(\frac{3}{4}cos\frac{\theta }{2}+\frac{1}{4}cos\frac{3\theta }{2}\right);\\ \tau_{r\theta }=\frac{K_{I}}{\sqrt{2\pi r}}\left(\frac{1}{4}sin\frac{\theta }{2}+\frac{1}{4}sin\frac{3\theta }{2}\right) \end{array}$$

where $\sigma_{r}$ and $\sigma_{\theta }$ are normal stresses, $\tau_{r\theta }$ is shear stress near the crack tip, *r* is the radial direction, and *θ* is the circumferential direction.

The direct elastic crack results for relegation near the crack tip are [19]:

(4) $$\begin{array}{l} u_{r}=\frac{K_{I}}{4\mu }\sqrt{\frac{r}{2\pi }}\left[\left(2\kappa -1\right)cos\frac{\theta }{2}-cos\frac{3\theta }{2}\right];\\ u_{\theta }=\frac{K_{I}}{4\mu }\sqrt{\frac{r}{2\pi }}\left[-\left(2\kappa +1\right)sin\frac{\theta }{2}+sin\frac{3\theta }{2}\right] \end{array}$$

where $u_{r}$ and $u_{\theta }$ are deportations near the crack tip, *r* is the radial direction, *θ* is the circumferential direction, and *μ* is the shear modulus. The value for μ is 10^6^.

## 3. Finite Element Analysis

**Figure 1:**
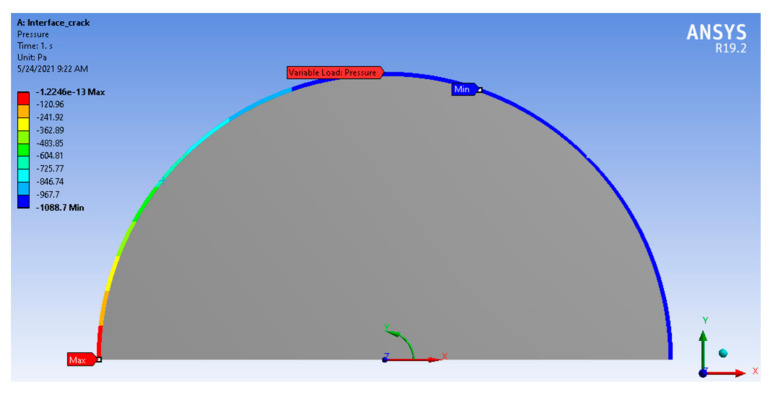
Variable shear load on arc boundary.

The nonlinear finite element software Ansys workbench was used in this study as it contained a nonlinear problem that could be answered by the sub-steps in the Ansys workbench. The applicable boundary conditions can be assessed in two ways. First, as this was a nonlinear problem, the computational process was divided into several sections in order to yield the correct result. This problem can be answered by using the sub-steps programme in the Ansys workbench. Second, we applied a varying shear load on the bow boundary, which cannot be directly set up by functions in the Ansys para-metric Design Language (APDL). To solve this problem, we used APDL to produce a command, and assigned this command to the bow in the Ansys workbench. The boundary was applied by varying shear load on bow boundary (see Figure 1).

### 3.1. Models

**Figure 2:**
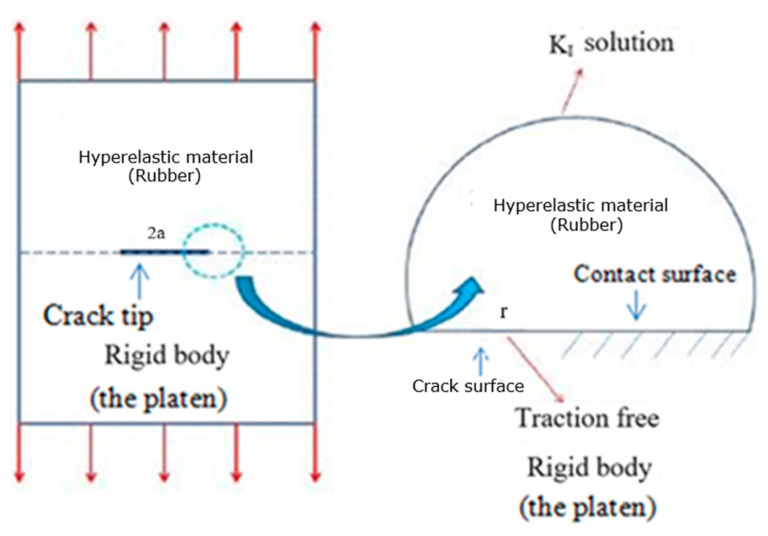
The semicircle with crack tip at center.

The main idea was to model a finite crack in a horizonless solid that was loaded under invariant traction at infinity. There was a traction free crack tip with a length of 2a at the interface. The region that was modeled is shown by a small circle around the crack tip. In the simulations, the model used a small semicircle around the crack tip with a radius of *r* *=* 5 (mm) (see Figure 2). The material parameters of the hyperelastic material models can be seen in Table 1.

#### 3.1.1. Neo-Hookean Model

The neo-Hookean model is generally considered to be a reduced polynomial form of the Mooney–Rivlin model. In the neo-Hookean model, the strain energy viscosity function can be written as [20]:

(5) $$\psi =C_{10}(\bar{I_{1}}-3)+\frac{1}{D}{\left(J-1\right)}^{2}$$

where *Ψ* is the strain energy viscosity function,$\;C_{10}$ and *D* are material parameters, *J* is the volume rate, and ${\bar{I}}_{1}$ is the first deviatoric strain invariant. The original shear modulus was given by $\mu_{0}$ = 2$C_{10}$, the original bulk modulus $K_{0}$ was related to *D* by $K_{0}=\frac{D}{2}$, where *D* = 0 for incompressible accoutrements. Therefore, Poisson’s rate can be expressed by $\upsilon =\frac{3K_{0}-2\mu_{0}}{6K_{0}+2\mu_{0}}$ where *Ψ* is the strain energy viscosity per unit volume in the disfigured configuration, and J is the volume rate. The first deviatoric strain invariant ${\bar{I}}_{1}$ can be expressed as $\bar{I_{1}}={\bar{\lambda }}_{1}^{2}+{\bar{\lambda }}_{2}^{2}+{\bar{\lambda }}_{3}^{2}$, where ${\bar{\lambda }}_{i}(i=1,2,3)$ is the deviatoric star stretch, and is related to the top stretch $\lambda_{i}$ by ${\bar{\lambda }}_{i}=J^{-\frac{1}{3}}\lambda_{i}$. The incompressible neo-Hookean model was the simplest hyperelastic native model we used, with one material parameter $C_{10}$, and was effective only in a fairly small strain range.

#### 3.1.2. Yeoh Model

The Yeoh hyperelastic model is a phenomenological model, and is applicable for large deformations of nearly incompressible, nonlinearly elastic accoutrements. The strain energy viscosity function is represented by [20]:

(6) $$\psi =C_{10}(I_{1}-3)+C_{20}{\left(I_{1}-3\right)}^{2}+C_{30}{\left(I_{1}-3\right)}^{3}$$

where *Ψ* is the strain energy viscosity function, $C_{10}$,$\;C_{20}$, and $C_{30}$ are material parameters,$\;I_{1}$1 is the first deviatoric strain invariant, and the original shear modulus $\mu_{0}$ is related to $C_{10}$ by $\mu_{0}$ = 2$C_{10}$. Although the Yeoh model is also a reduced polynomial form of the hyperelastic native model, it can easily predict the deformation of rubber in different distortion modes from data gained in just one distortion mode. It is also noteworthy that when *n* = 1, the Yeoh model reduces to the neo-Hookean model for incompressible accoutrements.

#### 3.1.3. Arruda–Boyce Model

The Arruda–Boyce hyperelastic native model, also known as the eight-chain model, was developed with reference to the statistical mechanics of a material with a square representative volume element containing eight chains alongside the slant directions. Applying the first five terms of the inverse Langevin function, the strain energy viscosity function for the incompressible Arruda–Boyce model is given by [20]:

(7) $$\psi =\mu \sum_{i=1}^{5}\alpha_{i}\beta^{i-1}(I_{1}^{i}-3^{i})$$

where *Ψ* is the strain energy viscosity function, μ is the shear modulus, $I_{1}^{i}$ is the first deviatoric strain invariant, $\beta =\frac{1}{\lambda_{m}^{2}}$, $\alpha_{1}=\frac{1}{2}$, $\alpha_{2}=\frac{1}{20}$, $\alpha_{3}=\frac{11}{1050}$, $\alpha_{4}=\frac{19}{7000}$, and $\alpha_{5}=\frac{519}{673,750}$. This model can directly predict the nonlinear mechanical behaviour of most elastic accouterments used in engineering with only two material parameters, the shear modulus μ, and the limiting network stretch (locking stretch) $\;\mu_{m}$. In this model, the original shear modulus $\mu_{0}$ is related to the shear modulus *μ* by $\mu_{0}=\mu \left(1+\frac{3}{5\lambda_{m}^{2}}+\frac{99}{175\lambda_{m}^{4}}+\frac{513}{875\lambda_{m}^{6}}+\frac{42,039}{67,375\lambda_{m}^{8}}\right)$, where the limiting network stretch $\lambda_{m}$ can be obtained by the limit chain $\lambda_{lim}$ as $\lambda_{m}=\sqrt{\frac{1}{3}\left(\lambda_{\lim}^{2}+\frac{2}{\lambda_{\lim}}\right)}$.

### 3.2. Meshing

**Figure 3:**
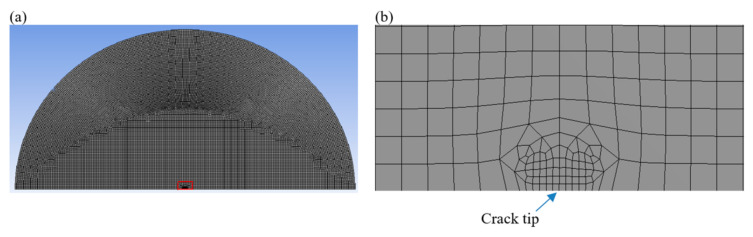
(**a**) Finite element model (coarse mesh) of the full model. (**b**) Semicircular finite element model with fine mesh at the crack tip.

The region of validity for the 1/r oddity is extremely small. Thus, it requires an extremely fine mesh near the crack tip. Still, the operation of large loads generates inordinate levels of mesh deformation. To overcome this deformation, the meshing was optimized using the so called “sub-modeling procedure”. We first ran an analysis using a coarse mesh on the full model figure (Figure 3a), and also reused the results from the coarse mesh model as boundary conditions for a sub-model with a finer mesh. The refined mesh model for the region around the crack tip is shown in Figure 3b. The element size (*e*) in the FE mesh near the crack tip was roughly *e* =$10^{-2}$, while the element size (e) in the FE mesh near the crack tip in the Yeoh model was approximately e=0.5.

### 3.3. Computation

The finite element simulations were carried out on the basis of the neo-Hookean, Yeoh, and Arruda–Boyce models using the parameters shown in Table 1, and a Poisson’s ratio $\nu_{0}$ = 0.49. The Poisson’s ratio of the elastomeric accoutrements used in the model was near 0.5, but in reality it is unlikely to actually equal 0.5 [25]. Similar elastomers show nonlinear reversible pliability for moderate distortions.

Note that a process-zone bounded by a circle of radius r should be generated around the crack-tip in such rubber-like accoutrements [1]. This core zone has been previously considered in the literature [26,27].

## 4. Experimental Work

**Figure 4:**
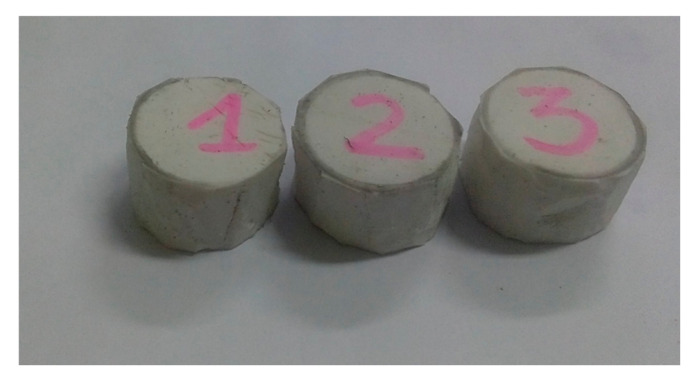
The three test specimens.

**Figure 5:**
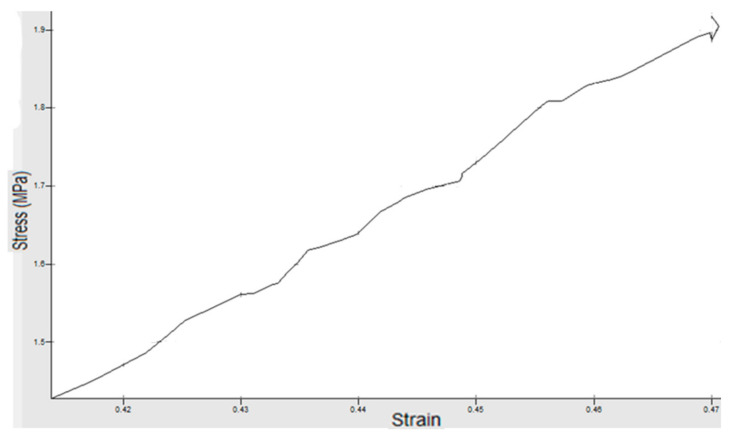
Piece 1 with speed *v* = 9mm/s and force *F* = 600 N.

**Figure 6:**
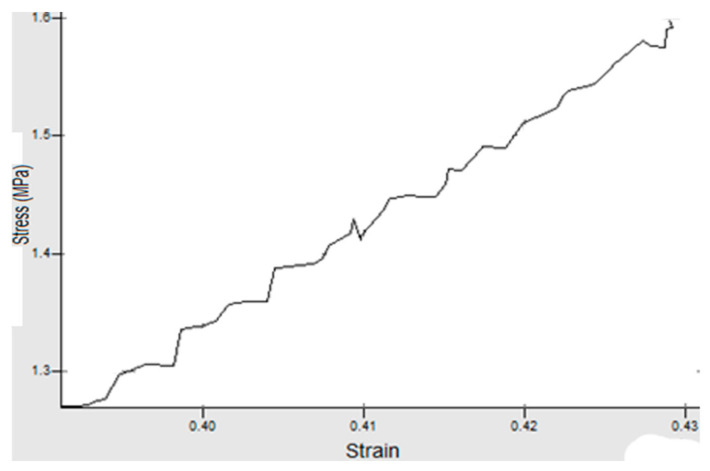
Piece 2 with speed *v* = 6 mm/s and force *F* = 500 N.

**Figure 7:**
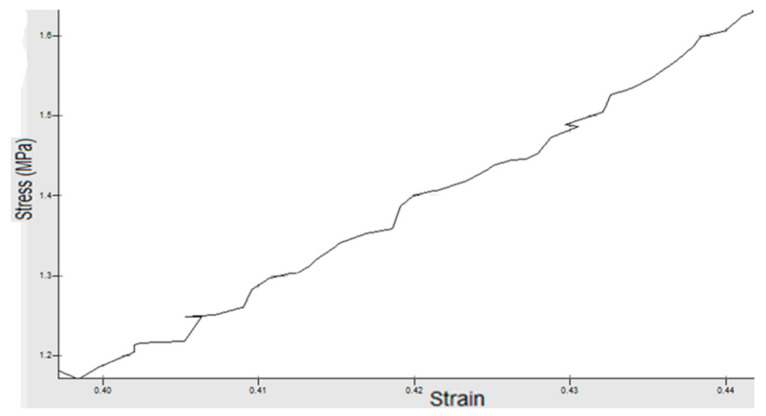
Piece 3 with speed *v* = 7 mm/s and force *F* = 510 N.

The test samples were cut out from the rubber jolt according to the ISO 23529 standard (see Figure 4). The specimens were tested under different speeds and forces on an LF2373 friction tester. The specimens 1, 2 and 3 were tested under speeds $v_{1}$($v_{1}=9\;\mathrm{mm}/s$),$v_{2}\left(v_{2}=6\;\mathrm{mm}/s\right)$, and $v_{3}\left(v_{3}=7\frac{\mathrm{mm}}{s}\right)$, and forces $F_{1}\left(F_{1}=600\;N\right)$, $F_{2}\left(F_{2}=500\;N\right)$, and $F_{3}\left(F_{3}=510\;N\right)$, respectively (see Figure 5, Figure 6, Figure 7). The maximum stresses and strains for Piece No. 1 were 1,865,143 Pa and 0.469864 (see Figure 5), for Piece No. 2 they were 1,543,659 Pa and 0.4289942844 (see Figure 6), and for Piece No. 3 they were 1,573,443 Pa and 0.441718 (see Figure 7), respectively.

## 5. Discussion

**Figure 8:**
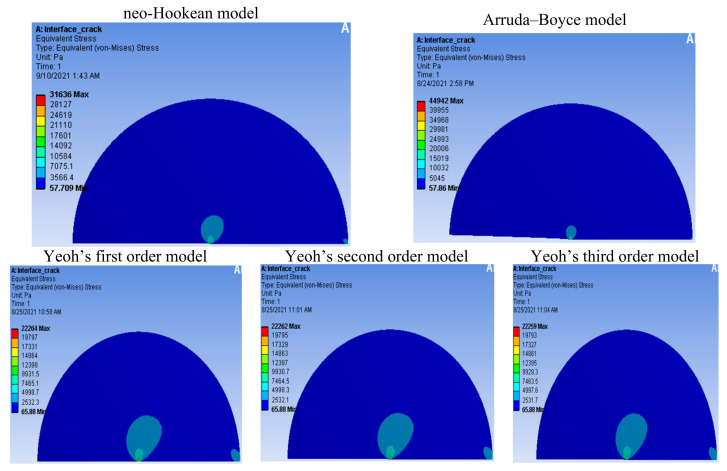
Distribution of the von Mises stress (Pa) during crack propagation.

**Figure 9:**
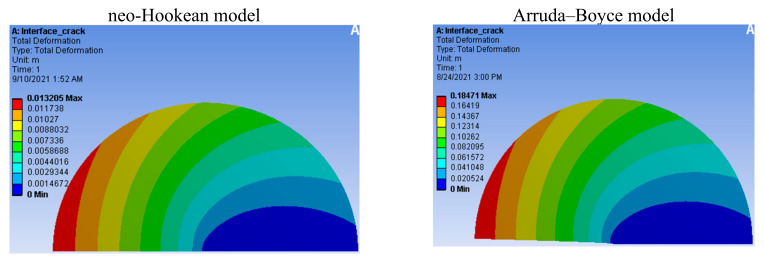

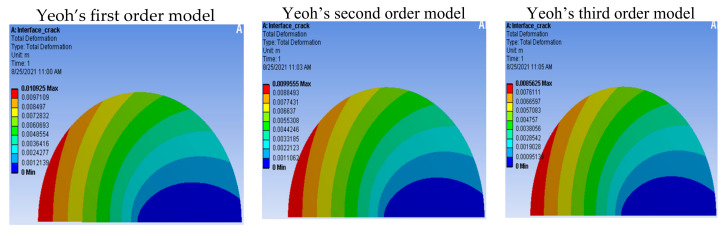
Contours of the deformation in the y direction (m).

We found the maximum stress in the Arruda–Boyce (44,942 Pa), neo-Hookean (31,636 Pa), Yeoh’s first-order (22,264 Pa), Yeoh’s second-order (22,262 Pa), and Yeoh’s third-order (22,259 Pa) models, as shown in Figure 8. From the existing theory concerning hyperelasticity [27], Figure 8 shows that the Yeoh models were better than the neo-Hookean model, and that the neo-Hookean model was better than the Arruda–Boyce model because although they have the same boundaries, the stress at the crack in the Yeoh models was smallest. In Figure 9, we can see the maximum deformation along the y-axis for the Arruda–Boyce model (0.18471 m), as well as the maximum stress in the neo-Hookean model (0.013205 m), Yeoh’s first-order model (0.010925 m), Yeoh’s second-order model (0.0099555 m), and Yeoh’s third-order model (0.0085625 m). According to the theory of hyperelastic materials [27], we could see that the Yeoh models were better than the neo-Hookean models, and that the neo-Hookean model was better than an Arruda–Boyce model. From the two cases shown above, we could see that Yeoh’s third-order model was the best model, as the models had the same boundaries, but the deformation by the crack in Yeoh’s third-order model was the smallest.

**Figure 10:**
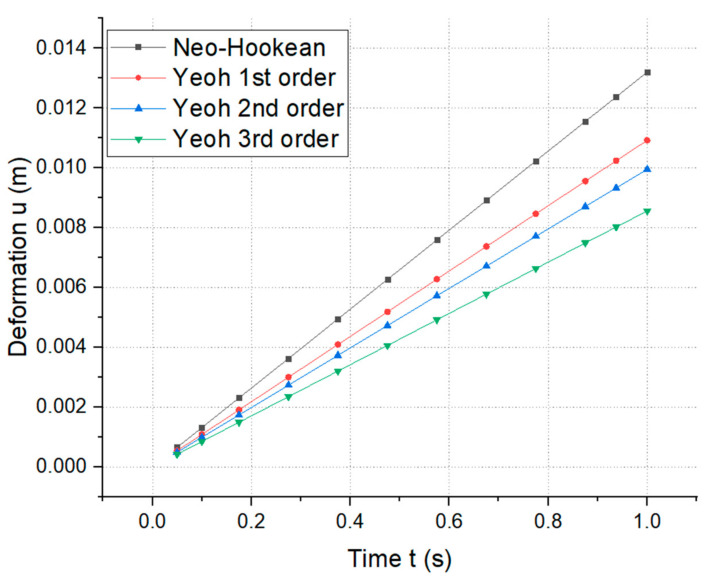
Variation of the normal deformation according to crack time in the hyperelastic material models.

**Figure 11:**
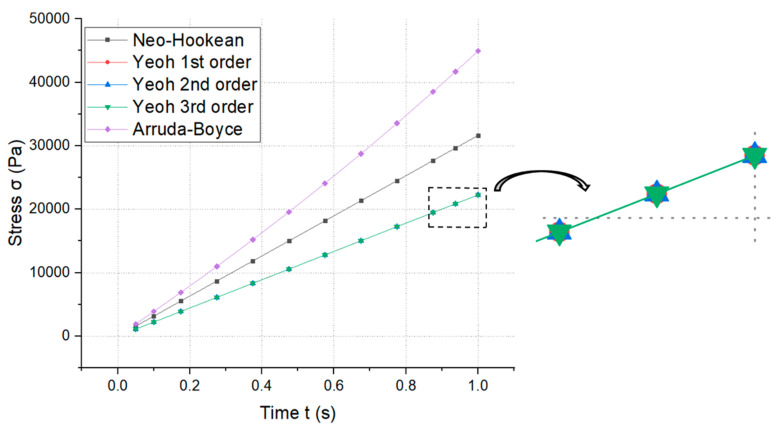
Variation in stress according to crack time in the hyperelastic material models.

Diagrams of the variation in the normal deformation according to the crack time in our hyperelastic material models are shown in Figure 10. At time t = 1 (s), we could see deformations in the neo-Hookean model (u=0.013205 m), Yeoh’s first-order model (u=0.01925 m), Yeoh’s second-order model (u=0.0099555 m), and Yeoh’s third-order model (u=0.0085625 m). According to hyperelastic theory [27], Figure 10 shows that the Yeoh models were better than the neo-Hookean model, and that Yeoh’s third-order model was the best model (at the same time t = 1 (s), and under the same boundary conditions) as the deformation at the crack in Yeoh’s third-order model was the smallest. Similar diagrams of the variation of stress according to crack time in the hyperelastic material models can be seen in Figure 11. At time t = 1 (s), we could see the maximum stress in the neo-Hookean model (σ=44,942 Pa), Yeoh’s first-order model (σ=22,264 Pa), Yeoh’s second-order model (σ=22,262 Pa), Yeoh’s third-order model (σ=22,259 Pa), and the Arruda–Boyce model (σ=22,770 Pa). Figure 11 shows that the Yeoh models were better than the neo-Hookean model and that the neo-Hookean model was better than the Arruda–Boyce model. Yeoh’s third-order model was the best model (at the same time t = 1 (s), and under the same boundary conditions) as the stress at the crack in Yeoh’s third-order model was the smallest.

**Figure 12:**
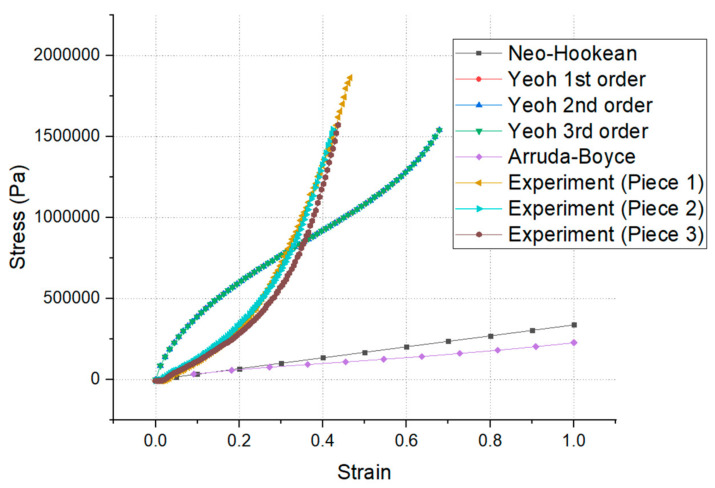
Comparison of the FEM and experimental curves for the hyperelastic materials.

The results of our stress–strain curve analyses are shown in Figure 12. We could see that from values of 0–20% strain, the curves of the neo-Hookean and Arruda–Boyce models were almost identical. We could also see that from the beginning of the test (0–5% strain), the curves produced by our experiments, those of the neo-Hookean model, and those of the Arruda–Boyce model were identical. However, our experiment’s curves and the Yeoh model’s curves converged at a certain point (30% strain for Piece No. 1 and 2, and 32% for Piece No. 3). At 30% strain, the FEM stress result in the neo-Hookean model was 101,350 Pa, the stress in the Arruda–Boyce model was 90,405 Pa, in Yeoh’s first-order model it was 769,490 Pa, in Yeoh’s second-order model it was 725,860 Pa, and in Yeoh’s third-order model it was 778,560 Pa (the largest value stress σ). The experimental stress result for Piece No. 1 was 702,325 Pa, for Piece No. 2 it was 673,949 Pa, and for Piece No. 3 it was 569,645 Pa. According to hyperelastic theory [27], Figure 12 shows that the Yeoh models were better than the neo-Hookean, and Arruda–Boyce models. Yeoh’s third-order model was the best model (the largest value stress σ).

## 6. Conclusions

In this paper, rubber’s stress–strain curves were determined. The parameters of a hyperelastic material model were determined using the above mentioned curves, the Ansys workbench, and APDL. At 30% strain, the FEM stress result in the neo-Hookean model was 101,350 Pa, the stress in the Arruda–Boyce model was 90,405 Pa, in Yeoh’s first-order model it was 769,490 Pa, in Yeoh’s second-order model it was 725,860 Pa, and in Yeoh’s third-order model it was 778,560 Pa (the largest value stress σ). The experimental stress result for Piece No. 1 was 702,325 Pa, for Piece No. 2 it was 673,949 Pa, and for Piece No. 3 it was 569,645 Pa. The finite element analysis results also indicated that the best results could be found in the Yeoh models. Our conclusions were obtained and validated via the use of numerical simulations and physical experiments.

It was found that the stress and strain could be used to estimate the value of nonlinear strain in the neo-Hookean, Yeoh, and Arruda–Boyce hyperelastic models. We could also see that the Yeoh models were better than the neo-Hookean model, and that the neo-Hookean model was better than the Arruda–Boyce model.

The obtained results could be useful for checking the validity of complex numerical schemes. The overall approach presented in this work represents a new path for characterizing the nonlinear mechanical actions of soft materials, for which no previous knowledge about the native response of a material is needed.

## Figures and Tables

**Table 1:** Material parameters of the models.

Hyperelastic Material Models	$\mathrm{Initial}\;\mathrm{Shear}\;\mathrm{Modulus}\;\mu_{0}$	Incompressibility Parameter lD	$\mathrm{Limiting}\;\mathrm{Network}\;\mathrm{Stretch}\;\lambda_{m}$
Neo-Hookean model	1.5012 × 10^5^ (Pa)	0 (Pa^−1^)	-
Yeoh 1st order model	6.0392× 10^5^ (Pa)	0 (Pa^−1^)	-
Yeoh 2nd order model	C1 = 6.6275 × 10^5^ (Pa)	D1 = 0 (Pa−1)	-
	C2 = −62,451 (Pa)	D2 = 0 (Pa−1)	
Yeoh 3rd order model	C1 = 7.7062 × 10^5^ (Pa)	D1 = 0 (Pa−1)	-
	C2 = −3.8631 × 10^5^ (Pa)	D2 = 0 (Pa−1)	
	C3 = 1.9685 × 10^5^ (Pa)	D3 = 0 (Pa−1)	
Arruda-Boyce model	23,157 (Pa)	1.5011 × 10^−7^(Pa^−1^)	5.3334

## Data Availability

Data sharing is not applicable.
